# UBQLN1 links proteostasis and mitochondria function to telomere maintenance in human embryonic stem cells

**DOI:** 10.1186/s13287-024-03789-y

**Published:** 2024-06-21

**Authors:** Shuang Zhao, Jie Li, Songqi Duan, Chang Liu, Hua Wang, Jiangtao Lu, Nannan Zhao, Xiaoyan Sheng, Yiwei wu, Yanjun Li, Baofa Sun, Lin Liu

**Affiliations:** 1 State Key Laboratory of Medicinal Chemical Biology, Nankai University, Tianjin, 300350 China; 2 Frontiers Science Center for Cell Responses, College of Life Science, Nankai University, Tianjin, 300071 China; 3 Experimental Animal Center, Nankai University, Tianjin, 300350 China; 4 Tianjin Union Medical Center, Nankai University, Tianjin, 300071 China

**Keywords:** UBQLN1, Telomere, Mitochondria, Ubiquitin-proteasome, Proteostasis, hESC differentiation

## Abstract

**Background:**

Telomeres consist of repetitive DNA sequences at the chromosome ends to protect chromosomal stability, and primarily maintained by telomerase or occasionally by alternative telomere lengthening of telomeres (ALT) through recombination-based mechanisms. Additional mechanisms that may regulate telomere maintenance remain to be explored. Simultaneous measurement of telomere length and transcriptome in the same human embryonic stem cell (hESC) revealed that mRNA expression levels of *UBQLN1* exhibit linear relationship with telomere length.

**Methods:**

In this study, we first generated *UBQLN1*-deficient hESCs and compared with the wild-type (WT) hESCs the telomere length and molecular change at RNA and protein level by RNA-seq and proteomics. Then we identified the potential interacting proteins with UBQLN1 using immunoprecipitation-mass spectrometry (IP-MS). Furthermore, the potential mechanisms underlying the shortened telomeres in *UBQLN1*-deficient hESCs were analyzed.

**Results:**

We show that Ubiquilin1 (UBQLN1) is critical for telomere maintenance in human embryonic stem cells (hESCs) via promoting mitochondrial function. *UBQLN1* deficiency leads to oxidative stress, loss of proteostasis, mitochondria dysfunction, DNA damage, and telomere attrition. Reducing oxidative damage and promoting mitochondria function by culture under hypoxia condition or supplementation with N-acetylcysteine partly attenuate the telomere attrition induced by *UBQLN1* deficiency. Moreover, *UBQLN1* deficiency/telomere shortening downregulates genes for neuro-ectoderm lineage differentiation.

**Conclusions:**

Altogether, UBQLN1 functions to scavenge ubiquitinated proteins, preventing their overloading mitochondria and elevated mitophagy. UBQLN1 maintains mitochondria and telomeres by regulating proteostasis and plays critical role in neuro-ectoderm differentiation.

**Supplementary information:**

The online version contains supplementary material available at 10.1186/s13287-024-03789-y.

## Introduction

Telomere locates at the end of the linear chromosome and maintains chromosomal stability and genome integrity [1], which primarily maintained by telomerase and also can be elongated by alternative telomere lengthening of telomeres (ALT) [2–4]. The typical hallmarks of aging include genomic instability, telomere attrition, epigenetic alterations, loss of proteostasis, deregulated nutrient sensing, mitochondrial dysfunction, cellular senescence, stem cell exhaustion, and altered intercellular communication [5, 6]. Understanding of the intimate signaling connections among these hallmarks of aging would provide molecular insights into mechanisms of aging and facilitate intervention of aging and age-related diseases.

A hallmark of many age-related diseases is the dysfunction in protein homeostasis (proteostasis), leading to the accumulation of protein aggregates. In healthy cells, a complex proteostasis network, comprising molecular chaperones and proteolytic machineries and their regulators, operates to ensure the maintenance of proteostasis. Proteostasis is critical to protein quality control and regulated by the ubiquitin-proteasome system (UPS) which degrades a protein molecule via ubiquitination and proteasomal degradation [7]. Extraproteasomal ubiquitin receptors are thought to couple the two steps. The UBL-UBA family proteins, including Ubiquilin1 (UBQLN1), are ideally suited to do so and are regarded as the shuttling Ub receptors, which include the Ub-like (UBL) domain at the N terminus, the Ub-associated (UBA) domain or domains at the C terminus and STI chaperone-like regions in the central region [8, 9]. The human genome encodes four ubiquilin genes, *UBQLN1*, *UBQLN2*, *UBQLN3*, and *UBQLN4*, which encode structurally related and conserved proteins. UBQLN1 plays an important role in clearing mislocalized mitochondrial proteins upon cell stimulation, and its absence leads to suppression of protein synthesis [10].

*UBQLN1* genetic variants or dysfunction is linked to neurodegenerative diseases such as Alzheimer’s disease (AD) [11–13]. Pathogenesis of AD, Parkinson’s disease (PD) and other neurodegenerative diseases is associated with the nine biological hallmarks of aging [14]. Telomere shortening was also shown to parallel with Alzheimer’s disease development and valued an early biomarker of AD progression [15–18].

Interestingly, expression levels of *UBQLN1* are highly positively correlated with telomere length by simultaneous measurement of telomere length and RNA-seq in the human embryonic stem cells (hESCs) [19]. hESCs maintain high telomerase activity and sufficient telomere lengths for their self-renewal and differentiation capacity [20–26]. In addition to telomerase, telomere associated proteins such as TPP1 and TINF2 also are involved in telomere elongation and telomere length homeostasis in hESCs [27–30]. Other factors, such as mitochondrial dysfunction and reactive oxygen species (ROS) [31], can also lead to telomere shortening. Moreover, hESCs may provide an unlimited cell source for replacement in a number of aging-related neurodegenerative diseases such as Parkinson’s disease and Alzheimer’s disease as well as other neurological disorders including spinal cord injuries [32]. UBQLN1 recently has been shown to involve in DNA replication by interacting with RPA1 and shuttling it off from the replication fork and *UBQLN1* depletion leads to rapid telomere shortening in HeLa cells [33]. Our data shows that *UBQLN1* regulates telomere maintenance in human ESCs by promoting proteostasis and mitochondria functions.

## Materials and methods

### Cell culture

WA26 (obtained from WiCell Research Institute) hESCs were routinely maintained in the undifferentiated state in E8 medium [34] (A1517001, Life technologies) on Matrigel-coated (356,230, BD Bioscience) tissue culture plates with daily medium change and passaged every 3 to 4 days using 0.5 mM EDTA in PBS at a 1:1000 ratio with Rocki (Selleck) [19], and cultured at 37 °C in 5% CO_2_ in an atmospheric O_2_ concentration. HEF cells (commercially available) were cultured in high glucose DMEM plus 10% FBS with 1% penicillin and streptomycin.

### Generation of UBQLN1 knockout hESCs by CRISPR-Cas9 technology

pSpCas9(BB)-2 A-Puro (PX459, Addgene plasmid #48,139) was a gift from Feng Zhang. Guide RNAs were designed using the online design tool available at http://crispr.genome-engineering.org/. PX459 was digested with BbsI and then gel purified. Two pairs of oligos including targeting sequences were annealed, guide RNAs of *UBQLN1* were cloned into BbsI-digested PX459 and transfect into passage 28 WT WA26 hESCs with Human Stem Cell Nucleofector® Kit 1. Finally, WT and *UBQLN1* KO clone was picked and identified by PCR. Primers used for CRISPR/Cas9 experiments are designed as below:

- sgRNA1: CCTCGTGATCAGCCAGCTT.
- sgRNA2: AGTCATTCCAGTAGGGAT.

The sgRNAs for *TERC*^−/−^ hESCs were designed as below,

- CRISPR-hTERC-oligo-L-F: CACCGCCTTTATAAGCCGACTCGCC.
- CRISPR-hTERC-oligo-L-R; AAACGGCGAGTCGGCTTATAAAGGC.
- CRISPR-hTERC-oligo-R-F: CACCGGGGTGACGGATGCGCACGAT.
- CRISPR-hTERC-oligo-R-R: AAACATCGTGCGCATCCGTCACCCC.

### Telomerase activity assay (TRAP)

Telomerase activity was determined using Telo Chaser Telomerase assay kit (TLK-101, TOYOBO). Approximately 2.5 × 10^4^ cells from each sample were lysed, and heated at 70 °C for 10 min to serve as negative controls. PCR products of cell lysates were separated on nondenaturing TBE-based 12% polyacrylamide gel electrophoresis and visualized by ethidium bromide staining [35].

### Cell cycle analysis

Cells were harvested and then fixed in freshly prepared precooled 70% ethanol overnight at 4 °C. Cells were centrifuged at 1000 g for 5 min and stained with propidium iodide (PI, Sigma) which is pretreatment RNase A (Thermo Fisher) at 37 °C for 30 min. Cell cycle phases were determined by FACS analysis and the data were processed using ModFit LT.

### Immunofluorescence microscopy

hESCs were fixed in 4% paraformaldehyde in PBS at 4 °C for 10 min, and then permeabilized with 0.1% Triton X-100 in PBS for 10 min at room temperature, followed by blocking in solution containing 5% BSA and 0.1% Triton X-100 for 2 h at room temperature. Samples were incubated with the primary antibodies overnight at 4 °C. The primary antibodies included Oct4 (sc5279; Santa Cruz), p-ATM(Millipore) and γH2AX (05–636; Millipore). Appropriate fluorescein-labeled secondary antibodies at room temperature for 2 h. Goat Anti-Mouse IgG (H + L) FITC (115–095 − 003; Jackson), Goat Anti-Rabbit IgG (H + L) Alexa Fluor® 594 (111–585-003; Jackson) and Goat Anti-Mouse IgM Alexa Fluor® 488(A-21,042; Invitrogen), diluted 1:200 with blocking solution, were used. Samples were washed and DNA was then stained for 10 min with Hoechst 33,342 (H1398; MP), and placed in Vectashield mounting medium. Fluorescence was detected and imaged using a Zeiss Axio-Imager Z2 fluorescence microscope or confocal microscope. Immunofluorescence (IF)-FISH was performed using TelC-Cy3 (F1002; Pangene). Integrated fluorescence intensity was estimated using ImageJ software, and the threshold was defined using non-specific background staining fluorescence.

### Western blot

Cells were washed twice in PBS, collected and lysed in cell lysis buffer (NP40 + PMSF + cooktail) for western blot on ice for 30 min and then sonicated for 40 s at 60 amplitude at 2 s intervals. Samples were centrifuged at 10,000 g for 10 min at 4 °C, then supernatants transferred into new tubes. The protein concentration was measured by bicinchoninic acid assay, and then the samples boiled with SDS buffer at 100 °C for 5–10 min. no more than 10 µg proteins of each sample were resolved by 8% or 10% Bis-Tris SDS-PAGE and transferred to polyvinylidene difluoride membranes (ISEQ00010; Millipore). Non-specific binding was blocked by incubation in 5% non-fat dry milk (9999 S; CST) in TBS-T at room temperature for 2 h. Blots were then probed with primary antibodies by incubation overnight at 4 °C in 5% skim milk in TBST. Immunoreactive bands were then probed for 2 h at room temperature with appropriate horseradish peroxidase (HRP)-conjugated secondary antibodies, anti-Rabbit IgG-HRP (NA934V; GE Healthcare), or goat anti-Mouse IgG (H + L)/HRP (ZB-2305; ZSGB-BIO). Protein bands were detected by Chemiluminescent HRP substrate (WBKLS0500; Millipore).

### Telomere measurement by quantitative real-time PCR (T/S ratio)

Genomic DNA was extracted by traditional phenol: chloroform: isoamyl alcohol method and the ratio of 260 to 280 was between 1.8 and 2.1. Average telomere length was measured using qPCR assay. Each 20 µL reaction was performed as follows: 35 ng gDNA, 1 × SYBR Green master mix (QPK-201, TOYOBO), 250 nM telomere forward primer and 250 nM telomere reverse primer, or 36B4 primers (forward primer: CAGCAAGTGGGAAGGTGTAATCC, reverse primer: CCCATTCTATCATCAACGGGTACAA). The telomere signal (T) was normalized to the signal from single copy gene (S) human 36B4 to generate a T/S ratio indicative of relative telomere length according to a standard curve [36]. Three repeat reactions were performed for each sample.

### Telomere quantitative fluorescence in situ hybridization (Q-FISH)

Telomere length and function (telomere integrity and chromosome stability) was estimated by Q-FISH [37]. Cells were incubated with 0.3 µg/mL nocodazole for 6 h to enrich cells at metaphases. Chromosome spreads were made by a routine method. Metaphase-enriched cells were exposed to hypotonic treatment with 0.075 M KCl solution, fixed with methanol: glacial acetic acid (3:1) and spread onto clean and cold slides. Telomeres were denatured at 80 °C for 3 min and hybridized with cy3-labeled (CCCTAA) peptide nucleic acid (PNA) probe at 0.5 µg/mL. Chromosomes were counter-stained with DAPI. Fluorescence from chromosomes and telomeres was digitally imaged on a Zeiss Imager Z2 microscope with Rho/DAPI filters. We quantified the signal of telomere foci using TFL-TELO software and made a normal distribution map for each group.

### Immunofluorescence-telomere FISH

hESCs were subject to immunostaining using γH2AX antibody as described in Immunofluorescence microscopy section. Excessive primary and secondary antibody was washed with PBS, stained nucleus by DAPI. Then cells were fixed in 4% formaldehyde for 5 min, dehydrated with 70%, 95% and 100% ethanol for 5 min each step. At last, incubated slide with Cy3-labeled telomeric PNA probe as described in Q-FISH section. Fluorescence was imaged using Zeiss Imager Z2 fluorescence microscope. The exposure time for γH2AX and Telomere were 1000 ms and 1500 ms respectively. Each group had no less than three repeats. Immunofluorescence (IF)-FISH was performed using TelC-Cy3 (F1002; Pangene) as previously described [38].

### Flow-FISH analysis of telomeres

Flow-FISH analysis of telomeres was performed as described previously [39]. Cells suspension was fixed with 70% alcohol for 10 min at 4 °C, and dehydration in 85% alcohol and 100% alcohol. Telomeres were denatured at 80 °C for 3 min and hybridized with Cy3-labeled (CCCTAA)3 peptide nucleic acid (PNA) probe at 0.5 mg/mL (F1002, Panagene, Korea). Then cells were shaken and washed three times and FACS analysis performed using a Flow Cytometer (BD Biosciences).

### Telomere restriction fragment (TRF) analysis

The average terminal restriction fragments (TRF) length of hESCs was determined according to the commercial kit (TeloTAGGG Telomere Length Assay, 12,209,136,001; Roche Life Science). Genomic DNA was extracted by traditional phenol: chloroform: isoamyl alcohol method. 2 µg DNA was digested with Hinf1 and Asc 1 overnight at 37 °C. The DNA fragments separated by 0.7% agarose gel for 2.5 h at 100 V in 0.5 × TBE buffer. Gels were denatured, neutralized, and transferred to nylon membrane (RPN2020B; GE Healthcare) for 24 h. The membrane was hybridized with digoxigenin (DIG)-labeled telomere probe at 42 °C overnight and incubated with anti-DIG-alkaline phosphatase antibody for 4 h. Telomere signal was detected by chemiluminescence after adding substrate solution on membrane. All TRF experiments were repeated for at least three times.

### ROS measurement

To detect intracellular ROS level, we used ROS-sensitive probe H2DCFDA, which was dissolved in DMSO to obtain a 10 mM stock solution and further diluted by 1:2000 ratio before use. For flow cytometry analysis, cells were incubated with 5 µM H2DCFDA in PBS in the dark for 30 min at 37 °C, then harvested with 0.5 mM EDTA solution, suspended in fresh PBS and immediately analyzed with flow cytometer. Cells were incubated with 5 µM H2DCFDA in PBS in the dark for 60 min at 37 °C [40], then washed with PBS and incubated with DAPI for 10 min. Samples were imaged using Axio-Imager Z2 Fluorescence Microscope (Carl Zeiss).

### Aggresomal detection

The cells were grown directly on glass slides, to a ~ 80% confluency. Positive control cells were prepared by incubating with the proteasome inhibitor MG132 (5 µM) for 18 h. The cells were carefully washed twice with PBS and excess PBS removed, then fixed with 4% formaldehyde for 30 min at room temperature. After being washed with PBS, the cells were permeabilized for 15 min. Samples were washed twice with PBS and incubated with dual detection reagent (ENZO, ENZ-51035-0025) for 30 min at room temperature, followed by washing and staining with DAPI. The samples were placed under coverslip on microscope slide, and imaged using a confocal microscopy. Standard rhodamine filter set was used for imaging the cell aggresome signal and a DAPI filter set for imaging the nuclear signal.

### Teratoma assay

WT and *UBQLN1*^–/–^ hESCs at passage 15 were cultured separately to a ~ 80% confluency. Then 10^6^ hESCs per site were injected into 6-week-old immunodeficient nude mice. Four mice were injected for each hESC line. One month after injection, the mice were humanely sacrificed, and the teratomas were excised, fixed in 4% paraformaldehyde at 4 °C overnight, dehydrated in gradient ethanol (70%, 85%, 95%, 100%) and xylene, and the incubation time for dehydration is based on tissue size, then embedded in paraffin, and sectioned for histological examination by H&E staining.

For H&E staining, sections were deparaffinized twice in xylene (each for 5 min) and rehydrated in gradient ethanol (100%, 85%, 70%, each for 5 min), stained with hematoxylin for 4 min, washed in ddH2O for 5 min, treated with 1.5% hydrochloric acid-75% ethanol for 4 s, washed in ddH2O for 5 min followed by PBS for 2 min, then stained with eosin for about 20 s, then dehydrated in gradient ethanol (70%, 85%, 95%, 100%) and xylene, and placed in xylene and neutral resin mounting medium. All experimental procedures were processed at room temperature.

### RNA-sequencing and analysis

The RNA was extracted using the QIAGEN RNA extraction Kit (74,134) following the manufacturer’s protocol, and RNA-seq was conducted using Illumina Sequencing (Novogene, China). Quality control of raw sequence data was performed with trim-galore (v0.6.5), followed by alignment to the human genome hg38 using hisat2 (v2.2.1) [41]. Read counts per gene were calculated using featureCounts (v2.0.6) with default parameters [42]. Differential gene expression analysis utilized the DESeq2 package (v1.26.0) [43] with a cut off log2FC > 1 and p-value < 0.05. Gene Ontology (GO) enrichment analysis of differentially expressed genes was carried out using the clusterProfiler R package (v4.8.1) [44] and DAVID [45]. Gene Set Enrichment Analysis (GSEA) was employed to identify predefined gene sets displaying statistically significant differences in expression patterns [46].

### Quantitative proteomic-MASS analysis

Sample was sonicated three times on ice using a high intensity ultrasonic processor (Scientz) in lysis buffer (8 M urea, 1% Protease Inhibitor Cocktail). (Note: For PTM experiments, inhibitors were also added to the lysis buffer, 3 µM TSA and 50 mM NAM for acetylation.) The remaining debris was removed by centrifugation at 12,000 g at 4 °C for 10 min. Finally, the supernatant was collected and the protein concentration was determined with BCA kit according to the manufacturer’s instructions. MS/MS was performed by PTM BIO. The peptides were subjected to NSI source followed by tandem mass spectrometry (MS/MS) in Q ExactiveTM Plus (Thermo) coupled online to the UPLC. The electrospray voltage applied was 2.0 kV. The m/z scan range was 350 to 1800 for full scan, and intact peptides were detected in the Orbitrap at a resolution of 70,000. Peptides were then selected for MS/MS using NCE setting as 28 and the fragments were detected in the Orbitrap at a resolution of 17,500. A data-dependent procedure that alternated between one MS scan followed by 20 MS/MS scans with 15.0 s dynamic exclusion. Automatic gain control (AGC) was set at 5E4. Fixed first mass was set as 100 m/z. The resulting MS/MS data were processed using Maxquant search engine (v.1.5.2.8) [47]. Tandem mass spectra were searched against human uniprot database concatenated with reverse decoy database. Trypsin/P was specified as cleavage enzyme allowing up to 4 missing cleavages. The mass tolerance for precursor ions was set as 20 ppm in First search and 5 ppm in Main search, and the mass tolerance for fragment ions was set as 0.02 Da. Carbamidomethyl on Cys was specified as fixed modification and acetylation modification and oxidation on Met were specified as variable modifications. Differential expression between groups was assessed using an independent two-sample T-test, ideal for our normalized data distribution. To mitigate the risk of false positives inherent in high-dimensional data, we applied a stringent false discovery rate (FDR) correction, setting the threshold at 0.01. Significant differential expression was defined by both CV < 0.1 and a minimum fold change of 1.2. All differentially expressed protein database accession or sequence were searched against the STRING database version 10.1 for protein-protein interactions. we fetched all interactions that had a confidence score ≥ 0.7 (high confidence).

### Co-immunoprecipitation (Co-IP) and liquid chromatography mass spectrometry (LC-MS)

Briefly, we harvested total protein from human ES cells (∼5 × 10^6^/reaction) stably transfected with UBQLN1 (Flag-tagged) expression vector in 10 cm dish and lysis with NETN buffer (20mM Tris (PH 8.0),1mM EDTA,100 mM NaCl, 0.5% NP40,100×PMSF and 100×Cocktail). Samples were vortexed for 30 S, and rotated at 4 °C for 30 min. Samples were then centrifuged at maximum speed for 10 min at 4 °C. Supernatant was collected with 50 µl as input. Lysis buffer was pre-cleaned by beads for three times (8 min each time) prior to incubating with antibodies. Immunoprecipitation was performed using anti-Flag antibody (Genescript, A00187-100) at 4 °C overnight. The immune complexes were precipitated by purified Protein G beads (Yeasen, 36405ES08) for 2–4 h at 4 °C. We boiled the beads with extracted proteins to separate it from proteins. The pull-down protein was then subjected to running in SDS-PAGE and extracted for liquid chromatography–mass spectrometry (LC/MS) analysis [48].

### Co-IP and ubiquitination analysis

Wild-type and *UBQLN1*^–/–^ hESCs transfected with 3Flag-ARF4 were harvested from 10 cm culture dish and resuspend with 50 µL PBS, then 150 µL 0.1% SDS was added. Samples underwent ultrasonication at 40% amplification for 15–20 times until the solution not viscous anymore. The protein sample was boiled at 100 °C for 20 min and 800 µL lysis buffer (20 mM Tris, PH 7.0),150 mM NaCl,1mM EDTA,1% Tritionx-100, 10% Glycerin) added. The next step was same as the above Co-IP experiment. Briefly, Immunoprecipitation was performed using anti-Flag antibody (Genescript, A00187-100) at 4 °C overnight. The immune complexes were precipitated by purified Protein G Mix beads (Yeasen, 36405ES08) for 2–4 h at 4 °C. Beads with extracted proteins were boiled to separate beads. After the pull-down protein was harvested with Flag antibody, the ubiquitination level of candidate protein ARF4 in wild-type and *UBQLN1*-deficient cells was assessed by western blot with Ubiquitin (linkage-specific K48) antibody. The Flag level was revealed by western blot and total protein as input (by Coomassie blue staining), which were used for quantification.

### Mice and care

Use of mice for this research was approved by the Nankai University Animal Care and Use Committee. All mice used in this study were taken care of and operated according to the relevant regulations. Mice were housed and cared in individually ventilated cages (IVCs) on a standard 12 h light:12 h dark cycle in sterile Animal Resources Center at Nankai University. Immunodeficient nude mice were purchased from Beijing Vital River Laboratory Animal Technology Co, Ltd. For testing cell differentiation ability experiments in vivo, the nude mice were quickly euthanized by standard cervical dislocation method without using chemical drugs and the teratoma removed for further analysis. The work has been reported in line with the ARRIVE guidelines 2.0.

### Embryoid body (EB) formation

To form hanging drop EBs, single cell drops (3000 cells/30 µL), which suspend with E8 media, hanging cultured on the lid of Petri dishes with 5 mL PBS at bottom. The dishes were incubated in 37 °C incubator overnight. On the second day EBs could be seen in each drop. The drops were continuously cultured and the aggregated EBs transferred into Corning Matrigel-coated 12-well plate at day 4 and continuously cultured to day 7.

### Statistical analysis

Statistical significances were analyzed by ANOVA or t-test using GraphPad prism 8. The differences between groups were considered significant when *P* < 0.05 (*), 0.01(**) or 0.001(***).

## Results

### Deletion of UBQLN1 shortens telomeres in hESCs

**Fig. 1:**
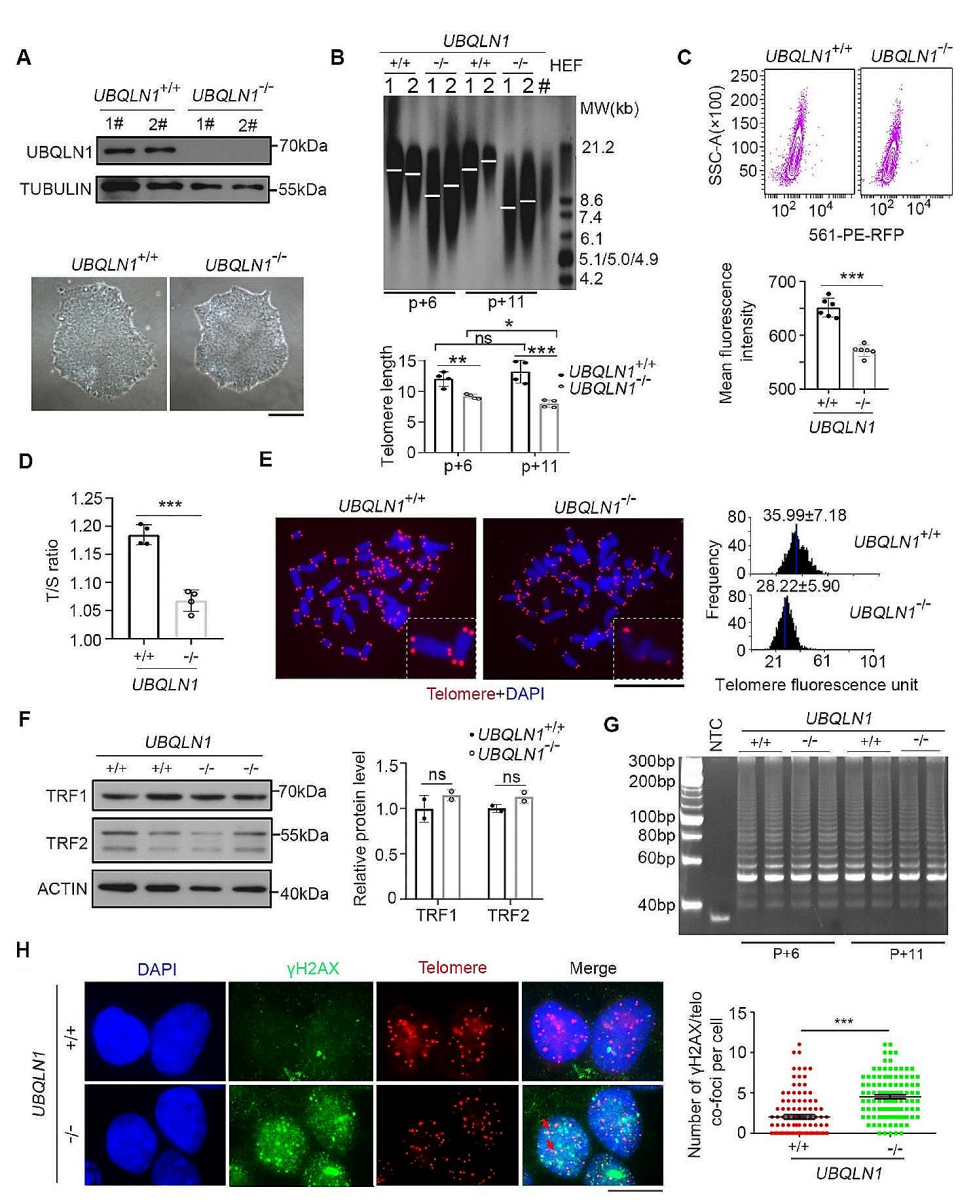
Dysfunctional telomere in *UBQLN1*^–/–^ hESCs **A** Upper panel, Western blot analysis of UBQNL1 protein level in *UBQLN1*^+/+^ and *UBQLN1*^–/–^ cells. Two clones were picked for *UBQLN1*^+/+^ and *UBQLN1*^–/–^ cells, respectively. Bottom panel, Representative cell morphology of *UBQLN1*^+/+^ and *UBQLN1*^–/–^ cells. Scale bar = 100 μm **B** Above: Telomere length distribution shown as TRF by Southern blot analysis of *UBQLN1*^+/+^ and *UBQLN1*^–/–^ cells at passage 6 and passage 11. HEF DNA was loaded as a control of short telomere. Below: Quantification of telomere length, *n* = 4. Statistical significances were analyzed by ANOVA. 1# and 2# represents two independent clones **C** Flow-FISH analysis by flow cytometry of telomere signal of *UBQLN1*^+/+^ and *UBQLN1*^–/–^cells. Right panel, Quantification of mean fluorescence intensity for Flow-FISH. *n* = 3 for each *UBQLN1*^+/+^ and *UBQLN1*^–/–^clone. Statistical significances were analyzed by t-test **D** Telomere measurement by quantitative real-time PCR (T/S ratio). 4 replicates are analyzed. Statistical significances were analyzed by t-test **E** Representative telomere Q-FISH images of *UBQLN1*^+/+^ and *UBQLN1*^–/–^hESCs. blue, chromosomes stained by DAPI; red dots, telomeres. Scale bar = 5 μm. Right panel, Relative telomere length was quantified and the average length ± SD is given in the upper **F** Western blot analysis of TRF1, TRF2 protein level in *UBQLN1*^–/–^ cells. β-ACTIN served as a loading control. Right panel, Quantification of the grayscale. Statistical significances were analyzed by t-test. The control group was set as unit 1 **G** Telomerase activity measured by TRAP assay. Lysis buffer served as negative control, *n* > 3 **H** IF-FISH images of telomere (red) and γH2AX (green). Colocalized foci are indicated by arrowheads. More colocalization of γH2AX/telomere foci occurred in *UBQLN1*^–/–^ hESCs. Scale bar = 10 μm. Right panel, Quantification of γH2AX/telomere co-foci number per cell. Statistical significances were analyzed by t-test. *n* > 100 cells were counted for each group *P < 0.05; **P < 0.01; ***P < 0.001. ns, no significant difference

Single cell RNA-seq analysis revealed that *UBQLN1* expression level is positively correlated with the telomere length in hESCs (Fig. S1A) [19]. To determine whether telomere length regulates *UBQLN1* expression, we generated telomerase-deficient *TERC*^–/–^ hESCs and compared the telomere length and *UBQLN1* expression levels with those of WT hESCs. Telomeres were shorter in *TERC*^–/–^ hESCs than in WT hESCs (Fig. S1B). By Western blot analysis, UBQLN1 protein levels were not decreased in *TERC*^–/–^ hESCs that had short telomeres (Fig. S1B), suggesting that telomerase deficiency and short telomeres do not down-regulate UBQLN1 expression. Hence, we generated *UBQLN1* knockout human ESCs using CRISPR/Cas9 method [49] and showed that deletion of *UBQLN1* consistently resulted in telomere shortening in repeated experiments by Southern blot (Fig. S1C; Fig. 1A, B) and the telomeres continuously shorten following passage without *UBQLN1* (Fig. 1B). Shorter telomeres found in both *UBQLN1*-knockout (*UBQLN1*^–/–^) hESC lines than in WT cells, were validated by flow-FISH [39] (Fig. 1C), T/S ratio (Fig. 1D), or Q-FISH method (Fig. 1E). These data indicate that UBQLN1 is required for telomere maintenance in hESCs.

hESCs typically express pluripotent transcriptional factor OCT4. By immunofluorescence, OCT4 protein expression was not altered by *UBQLN1* deficiency (Fig. S1D-E). The levels of shelterin complex components TRF1/2 and telomerase activity detected by TRAP assay [35], also remained similar between *UBQLN1*^–/–^ and WT hESCs (Fig. 1F, G). Analysis of cell cycle revealed that the percentage of S-phase was slightly reduced in hESCs without *UBQLN1*, compared with that of WT hESCs (Fig. S1F). Nevertheless, DNA damage was notably elevated in *UBQLN1*^–/–^ hESCs at late passage, as measured by immunofluorescence of γ-H2AX and p-ATM, compared with those of WT hESCs (Fig. 1H and Fig. S1G-H). Moreover, the frequency of micronuclei was increased in *UBQLN1*-deficient population (Fig. S1H). By simultaneous immunofluorescence of γ-H2AX and hybridization in situ (IF-FISH) analysis of telomeres [50], DNA damage at telomeres was increased in *UBQLN1*^–/–^ cells as evidenced by co-localization of γ-H2AX and telomere foci (Fig. 1H). In summary, *UBQLN1* deficiency leads to telomere shortening without affecting telomerase activity. Instead, UBQLN1 likely maintains telomeres by preventing telomere damage.

### Transcriptome and proteome analysis of *UBQLN1*^–/–^ hESCs

To understand potential signaling by which the loss of *UBQLN1* leads to telomere damage and shortening, we performed RNA-sequencing to reveal the molecular changes. Compared with WT hESCs, 81 genes were upregulated and 128 genes downregulated in *UBQLN1*^–/–^ hESCs (Fig. S2A, B). By GO and GSEA analysis, the results indicated that upregulated genes were related to biological processes including calcium-dependent cell-cell adhering, regulation of hemopoiesis and cell differentiation in spinal cord (Fig. S2C), while downregulated genes (with less significance) were enriched in mitochondria function, oxidation-reduction process as well as metabolic process(Fig. S2D-F). Integrative Genomics Viewer showed four representative oxidative-reduction related genes were downregulated in *UBQLN1*^–/–^ hESCs (Fig. S2G). The correlation analysis based on our published single cell data revealed that a positive but relatively low correlation between the expression of *UBQLN1* mRNA and these genes (Fig. S2H), further indicating that *UBQLN1* affects oxidation-reduction process. Reduction-oxidation reactions have an essential role in the protein structure maintenance through providing disulphide bonds and maintaining a proper redox environment for oxidative protein folding [51]. These RNA-seq data supports the notion that loss of *UBQLN1* may lead to altered transcriptome compromising mitochondria function and redox and this needs further validation.

**Fig. 2:**
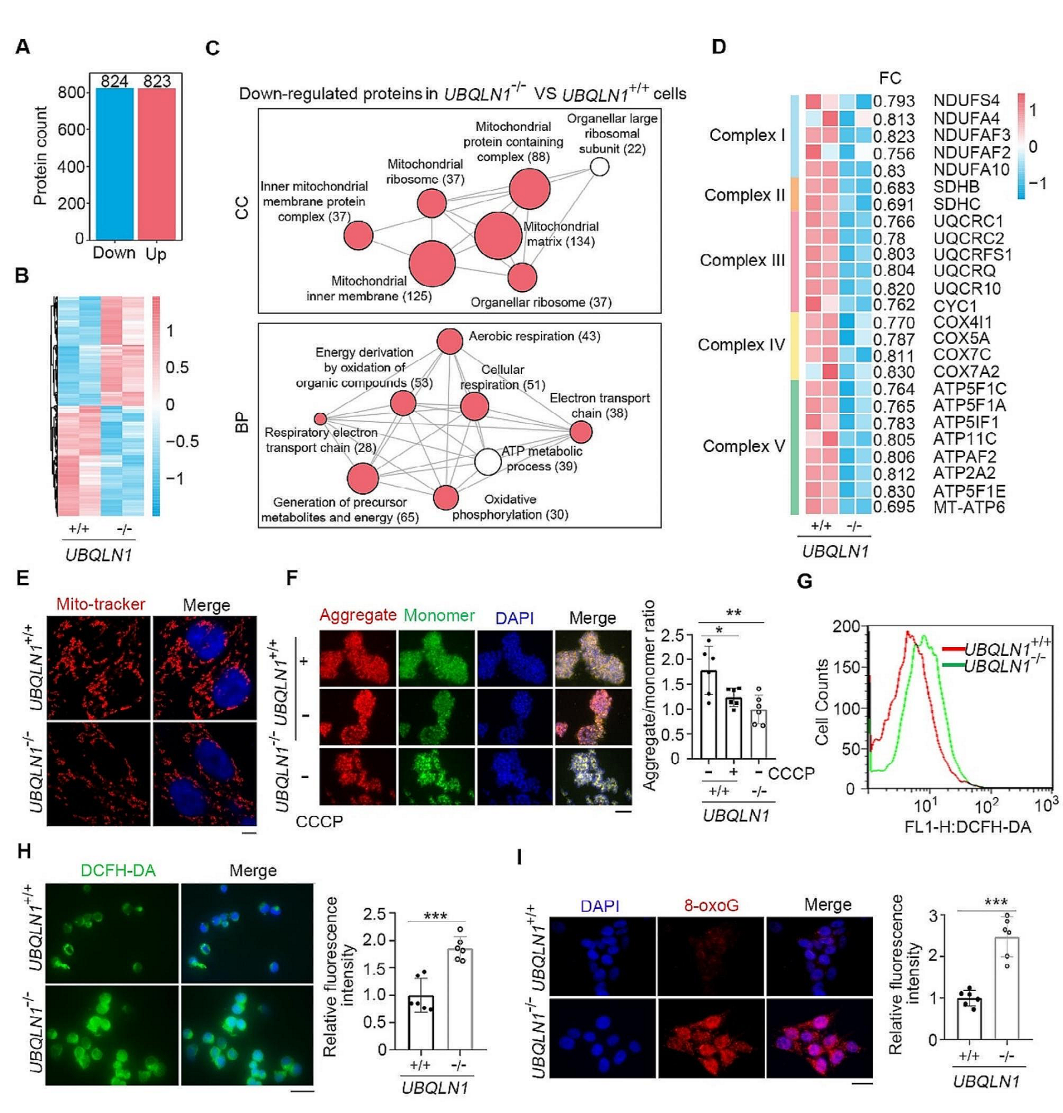
*UBQLN1* deficiency leads to mitochondrial dysfunction **A-B** The number (A) and the expression (B) of upregulated (CV < 0.1, FC > 1.2) and downregulated (CV < 0.1, FC  <1/1.2) proteins in *UBQLN1*^–/–^cells vs. *UBQLN1*^+/+^cells **C** Gene Ontology (GO) enrichment analysis of cellular component (GO-CC) and biological process (GO-BP) for the downregulated proteins in *UBQLN1*^–/–^ cells compared with *UBQLN1*^+/+^ cells. *P* < 0.05 **D** The heatmap shows the downregulated expression of electron transport chain componence of mitochondrial inner membrane. FC means fold change **E** Mitochondrial morphology and mass were visualized by staining with mito-tracker and captured by confocal microscope. Scale bar = 5 μm **F** Measurements of the MMP in *UBQLN1* KO hESCs using JC-1. *UBQLN1*^+/+^ cells treated with CCCP was used as positive control. Scale bar = 100 μm. Right panel, Quantification of aggregate/monomer ratio in *UBQLN1*^–/–^ cells and *UBQLN1*^+/+^. *n* = 3 for each *UBQLN1*^+/+^ and *UBQLN1*^–/–^ clone. Statistical significances were analyzed by ANOVA. **G** Flow cytometry analysis of ROS levels in *UBQLN1*^–/–^ hESCs **H** Immunofluorescence analysis of ROS levels in *UBQLN1*^–/–^ hESCs. Scale bar = 20 μm. Right panel, Quantification of mean fluorescence intensity, *n* = 3 for each *UBQLN1*^+/+^ and *UBQLN1*^–/–^ clone. Statistical significances were analyzed by t-test **I** Immunofluorescence analysis of oxidized guanine (8-oxoG) levels in *UBQLN1* KO hESCs. Scale bar = 20 μm. Right panel, Quantification of mean fluorescence intensity, *n* = 3 for each *UBQLN1*^+/+^ and *UBQLN1*^–/–^ clone. Statistical significances were analyzed by t-test. Three repeats of two different clones for each group. *P < 0.05; **P < 0.01; ***P < 0.001

UBQLN1 is known to involve in ubiquitin proteasome system-mediated protein degradation [9, 52]. To further understand the protein changes in *UBQLN1*-deficient hESCs, we performed quantitative proteomic-MASS analysis. The proteome of *UBQLN1*^+/+^ hESCs can clearly distinguish from that of *UBQLN1*^–/–^ hESCs by PCA analysis (Fig. 2A). 823 proteins increased and 824 proteins decreased in *UBQLN1*-deficient hESCs compared with WT hESCs (Fig. 2B). 24% of decreased proteins are located in mitochondria (Fig. S3A). GO analysis showed that a large number of down-regulated proteins were enriched in mitochondria and mainly inner mitochondrial membrane protein complex (Fig. 2C). Correspondingly, these decreased proteins in *UBQLN1*^–/–^ cells were mainly related to the biological process of aerobic respiration and electron transport on respiration chain (Fig. 2C-D). The main decreased electron transport chain proteins revealed by mass spectrometry were validated by western blot (Fig. S3B). Together, the quantitative proteomic-MASS analysis further suggests possible roles of UBQLN1 in maintaining mitochondria function.

Protein-protein interaction analysis demonstrated that the down-regulated proteins constituted a network of mitochondrial function, while the up-regulated proteins concentrated on the ubiquitin-proteasome system (Fig. S3C), consistent with the basic function of UBQLN1 in ubiquitylation mediated protein degradation. These data provide evidence that UBQLN1 likely influences the mitochondrial functions by direct or indirect pathways. The reduced electron transport chain (ETC) component also may lead to mitochondrial dysfunction.

### *UBQLN1*deficiency compromises mitochondria function

It is known that 13 proteins are encoded by mitochondrial genome [53]. Seven mitochondria-encoded protein were identified and all except for MT-ND5 were expressed at lower levels in *UBQLN1*^–/–^ hESCs than in WT hESCs, based on our MASS data (Fig. S3D). This may also be evidence of organelle defect [54]. The total mtDNA copy number was also decreased after loss of *UBQLN1* (Fig. S3E), which may explain for the decreased mitochondria-encoded proteins. However, the ATP level was up-regulated despite impaired mitochondria function accompanied by *UBQLN1* deletion (Fig. S3F). It is to note that hESCs possess active glycolytic metabolism which can generate considerable ATP when compared to somatic cells [55], and presumably the increased ATP could be produced by anaerobic respiration independent of mitochondria function.

We compared the morphology of mitochondria by Mitotracker immunofluorescence and found nearly similar mitochondria morphology but slightly reduced quantity in *UBQLN1*^–/–^ hESCs compared with *UBQLN1*^+/+^ cells (Fig. 2E). Moreover, the mitochondrial membrane potential (MMP) measured by JC-1 [56–58] was also reduced in *UBQLN1*^–/–^ hESCs, like CCCP-induced reduction of MMP in WT hESCs (Fig. 2F), corresponding to decreased componence of ETC in inner membrane of mitochondria and dysfunctional organelle. The reactive oxygen species (ROS) level was notably increased in *UBQLN1*-deficient cells when compared with WT cells by either flow cytometric analysis or immunofluorescence (Fig. 2G, H). ROS produced by mitochondria can enter nucleus by free diffusion process, which has been frequently shown to damage DNA and telomere [31, 59]. Telomeric DNA is thought to be particularly susceptible to ROS-mediated cleavage and base modifications [60, 61]. ROS induced telomere damage is mainly mediated by oxidized guanine (8-oxoG), which can either prevent telomere elongation or even leading to telomere cleavage [62, 63]. Indeed, we observed remarkably increased level of 8-oxoG in the nucleus of *UBQLN1*^–/–^ cells compared to WT cells (Fig. 2I). Together, these data indicate that *UBQLN1*^–/–^ deficiency may compromise mitochondria function and leads to oxidative damage.

### N-acetyl-L-cysteine (NAC) or hypoxia mitigates telomere shortening in *UBQLN1*^–/–^ hESCs

**Fig. 3:**
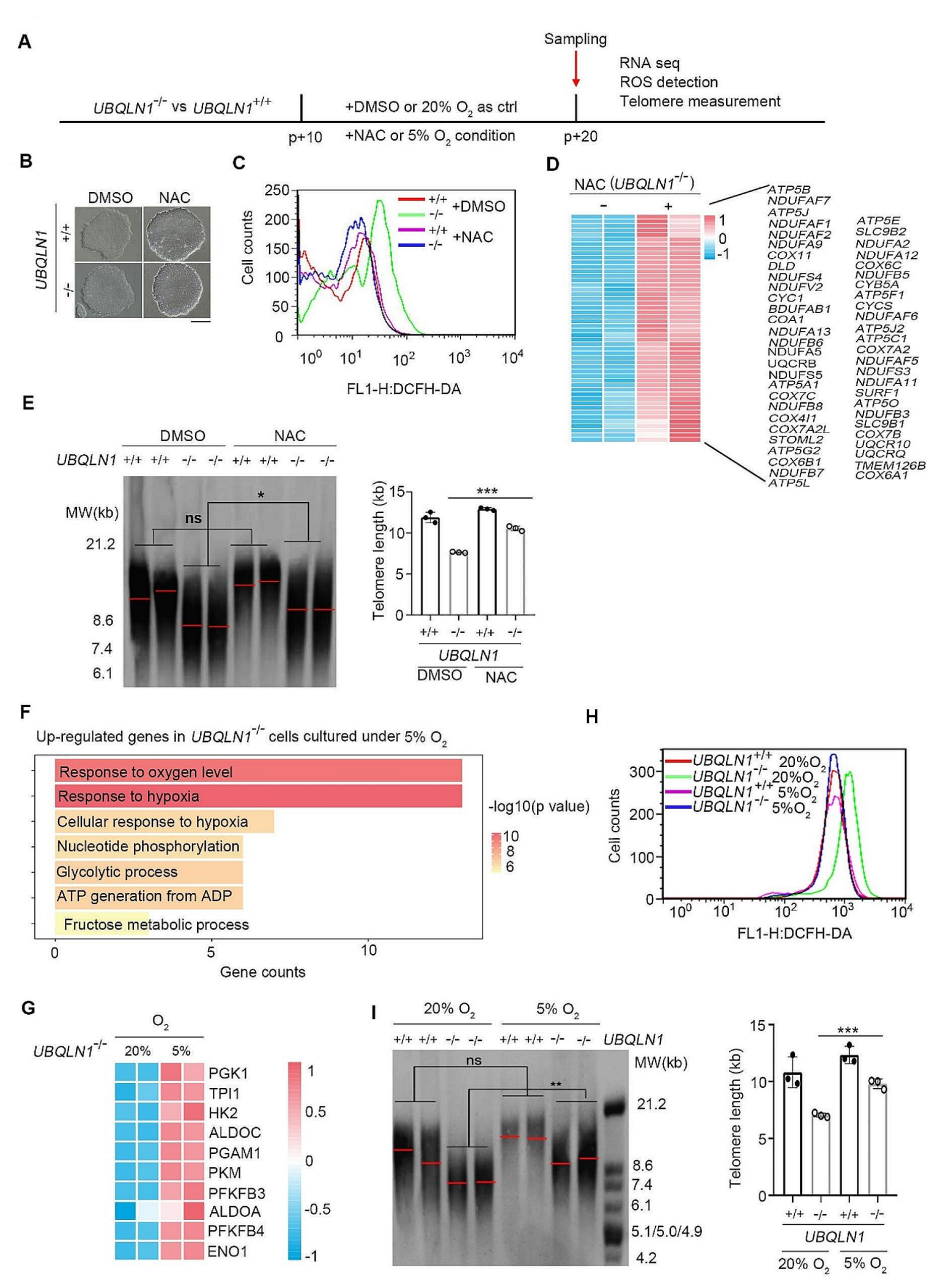
NAC or hypoxia alleviates telomere shortening in *UBQLN1*^–/–^ hESCs **A** Experimental scheme of cells treatment with NAC and low oxygen concentration for ten passages and harvest for RNA-seq, ROS detection and telomere measurement, DMSO treatment as negative control **B** Representative cell morphology of *UBQLN1*^+/+^ and *UBQLN1*^–/–^ cells cultured in medium supplemented with DMSO or NAC, respectively. Scale bar = 100 μm **C** Flow cytometry analysis of ROS levels in *UBQLN1*^–/–^ hESCs cultured in medium supplemented with DMSO or NAC, respectively. Reduced ROS signal can be seen in *UBQLN1*^–/–^ cells after treatment with NAC. **D** The heatmap shows genes in respiratory electron transport chain (ETC) of mitochondria upregulated in *UBQLN1*^–/–^ cells following NAC treatment **E** Telomere length distribution shown as TRF of *UBQLN1*^+/+^ and *UBQLN1*^–/–^ cells cultured in medium supplemented with DMSO or NAC, respectively. Right panel, Quantification of telomere length, *n* = 3. Statistical significances were analyzed by ANOVA **F** GO enrichment analysis of up-regulated genes when *UBQLN1*^–/–^ cells were cultured in 5% O_2_ when compared with in 20% O_2_ **G** Heatmap illustrating up-regulated oxidation-reduction associated genes of *UBQLN1*^–/–^ hESCs cultured in 5% O_2_ compared with 20% O_2_. Two biological replicates were analyzed per group. Genes with ≥ 2-fold expression changes, P-value < 0.05 were chosen for heatmap **H** Flow cytometry analysis of ROS levels in *UBQLN1*^–/–^ hESCs cultured in 20% O_2_ or 5% O_2_, respectively. Slightly reduced ROS signal can be seen in *UBQLN1*^–/–^ cells cultured in 5% O_2_ **I** Telomere length distribution shown as TRF of *UBQLN1*^+/+^ and *UBQLN1*^–/–^ cells cultured in 5% O_2_ and 20% O_2_, respectively. Right panel, Quantification of telomere length, *n* = 3. Statistical significances were analyzed by ANOVA ***P < 0.001

To test whether the ROS burst contributes to telomere damage and shortens telomeres in *UBQLN1*^–/–^ cells, we designed two experiments by employing permeable antioxidant NAC or by culture of the cells under hypoxia (5% O_2_) condition to reduce potential oxidative damage, followed by additional culture for 10 passages and measurement of telomere length (Fig. 3A). NAC is an effective ROS scavenger, which can decrease cellular ROS level with appropriate concentration [31, 64]. NAC can efficiently reduce ROS level in *UBQLN1*^–/–^ cells approximating that of *UBQLN1*^+/+^ hESCs and maintain normal ES cell clone morphology, like WT ESCs (Fig. 3B, C; Fig. S4A). The MMP also was recovered in *UBQLN1*^–/–^ cells after treatment with NAC (Fig. S4B). The 8-oxoG which may directly damage telomere DNA, was significantly reduced, accompanied by the decreased ROS level (Fig. S4C). Consistently, NAC also decreased γH2AX foci at telomeres in *UBQLN1*^–/–^ cells (Fig. S4D). Moreover, RNA-seq analysis revealed differential transcriptome between *UBQLN1*^–/–^ cells treated with and without NAC. Interestingly, many of reduced components of mitochondria in *UBQLN1*^–/–^ cells by MASS analysis as well as reduced expression of genes associated with oxidation-reduction process were restored at transcriptional levels following treatment with NAC (Fig. S4E, F). Also, genes in ETC of mitochondria were upregulated in *UBQLN1*^–/–^ cells following NAC treatment (Fig. 3D). These results suggest that in addition to its function as a ROS scavenger, NAC also can restore the mitochondria function by recovering the gene expression that is altered following *UBQLN1* deletion.

Furthermore, we examined the telomere length after treatment with NAC for additional 10 passages, compared with DMSO treatment served as vehicle control. NAC alleviated telomere shortening of *UBQLN1*^–/–^ cells (Fig. 3E). Hence, NAC recovers mitochondria functions as demonstrated by RNA-seq, ATP and MMP, reduces ROS and partly prevent telomere shortening of *UBQLN1*^–/–^ cells following continuous cultivation.

Additionally, we cultured ESCs under hypoxia (5% O_2_) and compared with conventional 20% O_2_ culture conditions. Analysis of the cell cultures for 24 h by RNA-seq showed that the transcriptome differed under the two culture conditions. Notably, the up-regulated genes in *UBQLN1*^–/–^ cells cultured under low versus normal O_2_ concentration were enriched in “glycolytic process” as well as “response to hypoxia” (Fig. 3F). Low O_2_ also improved the oxidation-reduction process weakened by the loss of *UBQLN1* (Fig. 3G). Compared to those under 20% O_2_, *UBQLN1*^–/–^ cells cultured under 5% O_2_ exhibited elevated MMP and declined levels of ROS and 8-oxoG (Fig. 3H; Fig. S5A-C). Elevated number of γH2AX co-localized with telomeres in *UBQLN1*^–/–^ cells under 20% O_2_ was noticeably reduced by cultures under hypoxia (Fig. S5D). Reduced telomere damage corroborates the lower 8-oxoG levels by 5% O_2_ shown above.

Furthermore, we compared the telomere length by TRF of *UBQLN1*^–/–^ cells cultured under low O_2_ condition for 10 passages with that of the control cultures under 20% O_2_. Low O_2_ culture alleviated telomere shortening in *UBQLN1*^–/–^ cells (Fig. 3I). Transcriptome of *UBQLN1*^–/–^ hESCs separated well from that of *UBQLN1*^+/+^ hESCs by Principal Components Analysis (PCA) (Fig. S5E). Transcriptome of *UBQLN1*^–/–^ cells cultured under 5% O_2_ was closer to that of *UBQLN1*^+/+^ in both first and second principal component (Fig. S5E). By integrated analysis, genes up-regulated in both NAC and 5% O_2_ conditions were enriched in the pathway of hypoxia response (Fig. S5F), indicating the similar switch of metabolism pattern.

Together, 5% O_2_ cultures also reduce ROS, recover mitochondria functions and attenuate telomere shortening induced by *UBQLN1* deficiency.

### UBQLN1 prevents the ubiquitinated proteins from overloading mitochondria

**Fig. 4:**
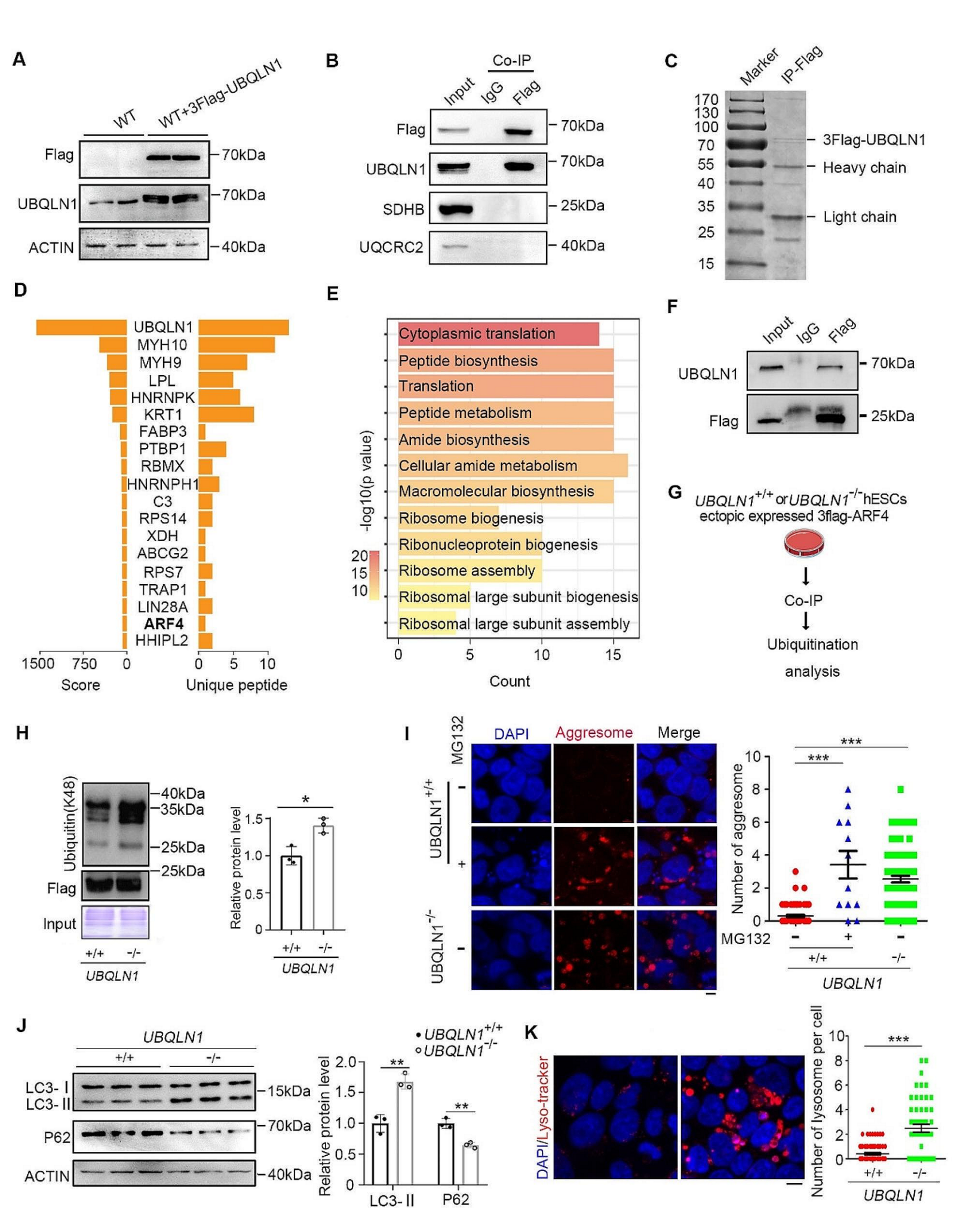
UBQLN1 maintains proteostasis **A** Identification of 3Flag-UBQLN1 cell lines for Co-IP **B** Validation by Co-IP of Non protein interaction between UBQLN1 and mitochondria component. IgG served as control **C** The Coomassie blue staining of pull-down protein sample by Flag antibody. The band of 3Flag-UBQLN1 can be seen **D** The bar plot shows UBQLN1-interaction proteins in MS/MS results, with UBQLN1 itself possessed the highest score **E** GO enrichment analysis of UBQLN1-interaction proteins **F** Validation by Co-IP of the interaction between ARF4 and UBQLN1. 3Flag-ARF4 cell line was used to pull-down the protein **G** Experimental scheme of ubiquitination analysis for ARF4 **H** Ubiquitin(k48) levels of ARF4 was analysis by western blot and quantified by image J after pull-down by flag antibody. Flag served as a loading control. Statistical significances were analyzed by t-test **I** Protein aggresome was visualized by staining and captured by confocal microscope. Right panel, quantification the number of visible aggresome per cell. Statistical significances were analyzed by t-test. Five fields were counted for each group. Scale bar = 5 μm **J** Western blot analysis of LC3 and P62 level in *UBQLN1* KO and control group. β-ACTIN served as a loading control, *n* = 3. Right panel, quantification of the grayscale, *n* = 3. Statistical significances were analyzed by t-test. The control group was set as unit 1 **K** Lysosome was visualized by staining with lyso-tracker and captured by confocal microscope. Scale bar = 5 μm. Right panel, number of visible lysosome in each cell of *UBQLN1*^–/–^ and *UBQLN1*^+/+^ cells. Statistical significances were analyzed by t-test. Five fields were counted for each group *P < 0.05; **P < 0.01; ***P < 0.001

To functionally investigate how UBQLN1 regulates mitochondria function, we searched for the UBQLN1-interacting proteins. We constructed the 3flag-UBQLN1 cell line (Fig. 4A). Unfortunately, we failed to see direct interactions of UBQLN1 with the mitochondria proteins such as SDHB and UQCRC2 that were reduced in *UBQLN1*^–/–^ cells shown above (Figs. 2C and D and 4B). It is possible that UBQLN1 regulates shuttling of many ubiquitinated proteins without forming a stable interaction enough with them to be recovered by co-IP. Then, we carried out Co-IP/MASS analysis (Fig. 4C), and found many UBQLN1-interacting proteins such as ARF4 and signal pathways that were upregulated, notably ribosome biogenesis and metabolisms implicated in translation and protein synthesis (Fig. 4D, E). That many of the upregulated protein following *UBQLN1* deficiency overlapped with UBQLN1-interacting proteins, such as RPS75b, MYH9, ARF4, PSMA6, EIF4A1 and RPL10, may suggest that UBQLN1 could be implicated in regulation of protein degradation for proteostasis.

We have identified ARF4 as an UBQLN1-interacting protein (Fig. 4F) as well as its increase in *UBQLN1*^–/–^ cells. By pull-down experiments combined ubiquitylation analysis, we observed ubiquitin modified ARF4 accumulated in *UBQLN1*^–/–^ cells (Fig. 4G and H). We also could see abundant protein aggresome accumulated in the cytoplasm of *UBQLN1*^–/–^ cells, in contrast to WT cells (Fig. 4I). Proteosome inhibitor MG132 effectively promoted the aggresome, which may serve as a positive control, further supporting proteasome degradation deficiency in *UBQLN1*^–/–^ cells. The aggresome appears to be the product of the most extreme form of protein aggregation that is observed in cells whose proteasome function is chronically blocked [65]. When the “Garbage proteins” failed to be degraded by proteasome and reside largely in cytoplasm, many of them can be carried to mitochondria [66] and lead to mitophagy as well as elevated ROS [67]. Furthermore, increased mitophagy as shown by marker LC3-II (reflecting autophagic activity) and decreased P62 protein was detected in *UBQLN1*^–/–^ cells, compared to WT cells (Fig. 4J), coincident with more emerging lysosomes (Fig. 4K). These data show that UBQLN1 is required to clear the ubiquitinated proteins and maintain functional mitochondria.

**Fig. 5:**
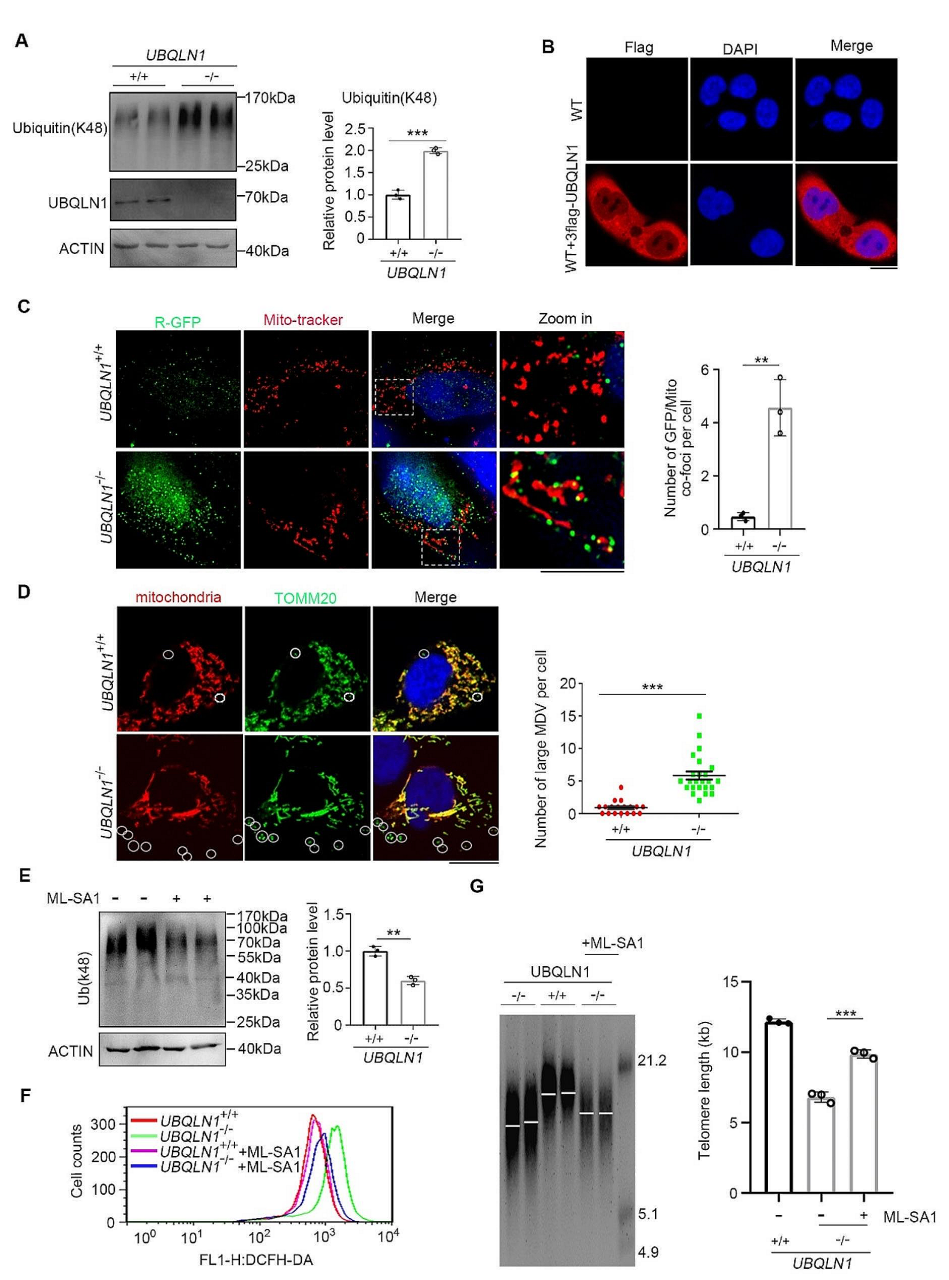
*UBQLN1* deficiency leads to ubiquitinated protein accumulation to overload mitochondria **A** Total ubiquitinated protein level in *UBQLN1*^–/–^ and control group. β-ACTIN served as a loading control. Right panel, quantification of the grayscale, *n* = 3. Statistical significances were analyzed by t-test. The control group was set as unit 1 **B** Immunofluorescence image indicate UBQLN1(Tagged by Flag) mainly locates at cytoplasm and also with some in nucleus. Scale bar = 10 μm. WT hESCs without transfection of flag tagged UBQLN1 was used as a negative control **C** Immunofluorescence analysis of Ub-R-GFP location in *UBQLN1* KO and *UBQLN1*^+/+^ hESCs. Right panel, quantification the number of GFP/Mitochondria co-foci per cell, *n* = 3. Scale bar = 10 μm. Statistical significances were analyzed by t-test. Three fields were counted for each group **D** Immunofluorescence analysis of TOMM20^+^ MDV in *UBQLN1* KO and *UBQLN1*^*+/+*^ hESCs. Right panel, quantification the number of large MDV per cell. Scale bar = 10 μm. Statistical significances were analyzed by t-test. Ten fields were counted for each group **E** Analysis of total ubiquitinated protein level after treatment of ML-SA1. Right panel, quantification of the grayscale, *n* = 3. Statistical significances were analyzed by t-test. The control group was set as unit 1 **F** Flow cytometry analysis of ROS levels in *UBQLN1*^–/–^ hESCs cultured in medium supplemented with ML-SA1 or not, respectively. Slightly reduced ROS signal can be seen in *UBQLN1*^–/–^ cells after treatment with ML-SA1 **G** Telomere length distribution shown as TRF of *UBQLN1*^+/+^ and *UBQLN1*^–/–^ cells cultured in medium supplemented with ML-SA1 for 10 passages (from passage 10 to 20) or NOT, respectively. Right panel, quantification of telomere length, *n* = 3 repeated experiments. Statistical significances were analyzed by ANOVA **P < 0.01; ***P < 0.001

By further analysis of the proteome data, the upregulated proteins in *UBQLN1*^–/–^ cells showed the enrichment in autophagy and ubiquitin-mediated proteolysis by GO and KEGG analysis (Fig. S6A, B). It is likely that *UBQLN1* deficiency compromises protein degradation in proteasome by abrogating ubiquitin-proteasome system, consistent with UBQLN1’s function as a shuttle to carry ubiquitin modified protein to proteasome followed by degradation. In addition to the ubiquitin associated activity, the upregulated proteins were also enriched in multiple metabolisms, immune process, kinase activity as well as cell cycle (Fig. S6A, B). The aberrant up-regulation of these proteins also forebodes accumulation of mass proteins with destiny to degradation in *UBQLN1*^–/–^ cells. Lysine-48 (K48) linked polyubiquitin chains are well established as the canonical signal for proteasomal degradation [68]. Quantitative mass spectrometry analyses of intracellular ubiquitin linkages support this notion, as K48-polyubiquitin linkage rapidly accumulates when cells are treated with the proteasome inhibitor MG132 [69]. To validate our hypothesis, we detected that the ubiquitin (k48) level of total protein was increased in *UBQLN1*^–/–^ cells (Fig. 5A). UBQLN1 located mainly at cytoplasm and also with some in nucleus as tagged by flag (Fig. 5B), coincided with the distribution of ubiquitin modified proteins reported in a previous report [70].

To examine whether the ubiquitinated proteins are accumulated around the mitochondria, we infected Ub-R-GPF (a GFP- based reporter for ubiquitinated proteins) into *UBQLN1*^+/+^ and *UBQLN1*^–/–^ hESCs, based on the method described previously [66]. Notably, the accumulated GFP signals representative of ubiquitinated proteins supposedly with destiny to degradation were recruited near or even into mitochondria in *UBQLN1*^–/–^ cells, distinguishable from *UBQLN1*^+/+^ cells (Fig. 5C). Mitochondrial-derived vesicles (MDVs), which are enriched for the outer mitochondrial membrane (OMM) import receptor TOMM20 and cannot be stained by mitochondrial probes and lack most of respiratory chain component proteins, are implicated in diverse physiological processes—for example, mitochondrial quality control—and are linked to various neurodegenerative diseases [71]. TOMM20^+^ MDVs facilitate mitophagy in response to functional impairments [71]. In early studies, MDVs carrying matrix cargo were stimulated by mild oxidative stress and were mapped to deliver mitochondrial proteins within 1–3 h to multivesicular bodies/lysosomes for degradation, whereas mitophagy would follow at later times (after approximately 24 h) [72, 73]. We observed increased number of large TOMM20^+^ MDV in *UBQLN1*-deficient hESCs, compared with those of WT cells (Fig. 5D). Together with increased LC3-II and decreased p62, *UBQLN1* loss may lead to increased mitophagy, followed by increased ROS.

**Fig. 6:**
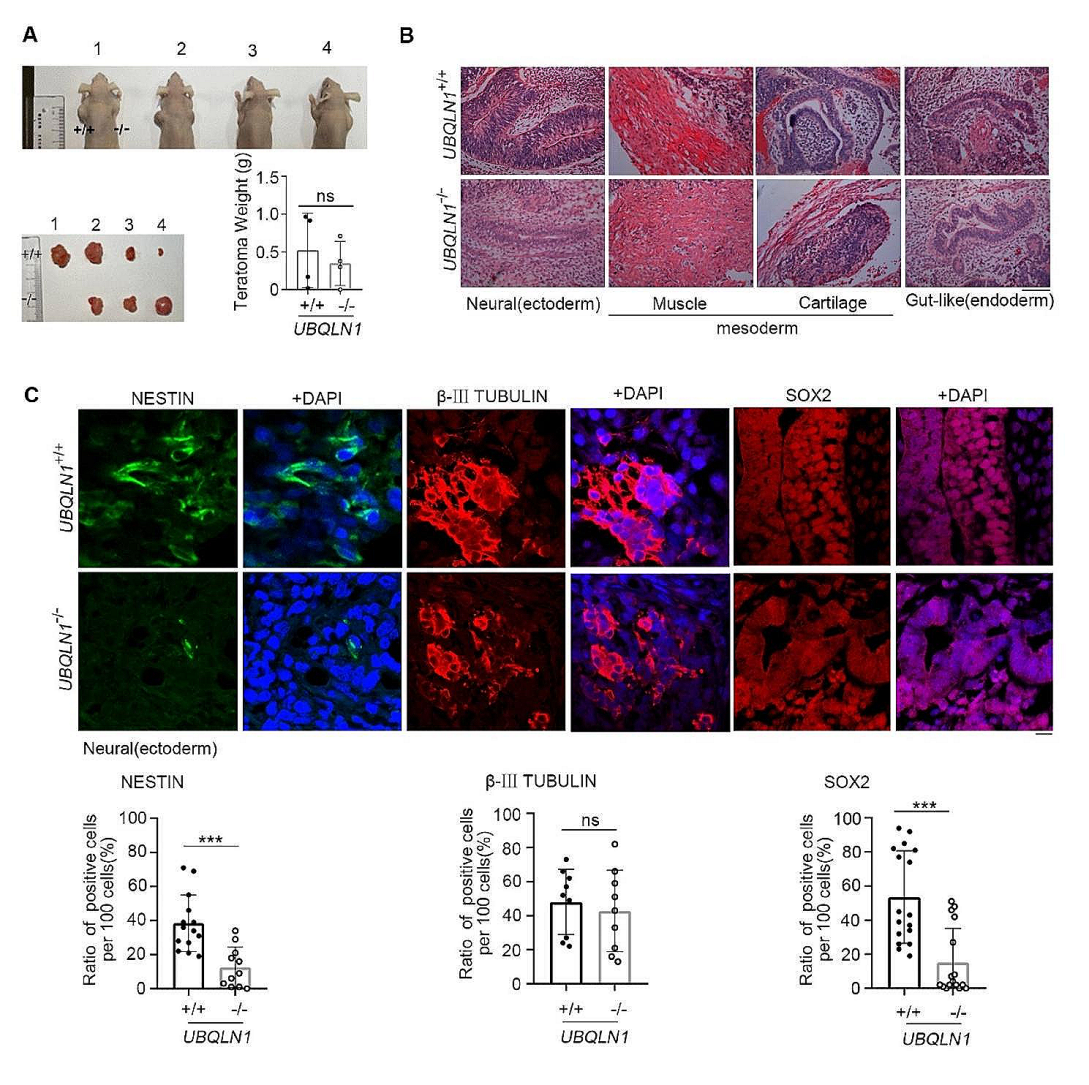
Neural differentiation defect resulting from *UBQLN1* deficiency A The teratoma formed by WT and *UBQLN1*^–/–^ cells after injection into immunodeficient nude mice. *n* = 4, Statistical significances were analyzed by t-test B H&E histology of the teratoma formed by WT and *UBQLN1*^–/–^ cells. The present picture indicates typical triple germ layer. Scale bar = 100 μm C Above: immunofluorescence analysis of neural ectoderm marker, NESTIN,β-III TUBULIN and SOX2. Bottom: Quantification of mean fluorescence intensity. Scale bar = 100 μm. Statistical significances were analyzed by t-test. Ten fields were counted for each group ***P < 0.001; ns, no significance

Treatment of *UBQLN1*^–/–^ cells with ML-SA1, which can activate lysosomal pathway by promoting lysosomal acidification and activity of the lysosomal enzymes [74], partly reduced accumulation of protein with ubiquitination modify (Fig. 5E) and also decreased ROS production (Fig. 6F). Notably, activation of lysosomal pathways by ML-SA1 treatment for 10 passages (from passage 10 to 20) reduced telomere shortening in *UBQLN1*^–/–^ cells during continuously passages (Fig. 5G).

These results are consistent with the notion that UBQLN1 maintains mitochondrial function and telomeres by regulating proteostasis.

#### UBQLN1 mutation leads to neural differentiation defect

We performed teratoma formation experiment to test the differentiation ability after injection of WT or *UBQLN1*^–/–^ hESCs into immunodeficient nude mice. *UBQLN1*^–/–^ hESCs can form teratoma at similar size without statistical differences compared with WT hESCs (Fig. 6A), even if *UBQLN1*^–/–^ hESCs possess shortened telomere, defective protein homeostasis and dysfunctional mitochondria. However, the teratoma formed from *UBQLN1*^–/–^ hESCs displayed deficient neural differentiation as indicated by H&E staining (Fig. 6B) and reduced specific neural markers, such as NESTIN and SOX2, but not β-III TUBULIN (Fig. 6C), while the development of mesoderm and endoderm was normal (Fig. 6B). Moreover, we also compared the embryoid body (EB) formation from WT or *UBQLN1*^–/–^ hESCs. Deficient EB differentiation ability was found in *UBQLN1*^–/–^ hESCs as to both the size and number (Fig. S7A). Furthermore, we also found reduced expression of neuronal marker SOX2 (Fig. S7C) but not β-III TUBULIN (Fig. S7B) in EBs differentiated from *UBQLN1*^–/–^ hESCs compared with WT hESCs. Hence, UBQLN1 plays an important role in neuronal differentiation of hESCs. The mechanisms of how UBQLN1 regulate neuronal differentiation could be complex and remains to be investigated in the future. Yet, our initial results based on *UBQLN1*^–/–^ hESCs may provide a useful model to investigate the role of UBQLN1 in neurogenesis. This result may also partly explain the consequences of *UBQLN1* mutation in the pathogenesis of neuron degenerative Alzheimer’s and Parkinson’s diseases.

## Discussion

Our work firstly uncovers the ability of UBQLN1 in maintaining telomere and links the UBQLN1 involved ubiquitin proteasome system to mitochondria function as well as telomere stability in human ESC model. Artificial stress to the mitochondria that disrupts mitochondria functions has been shown to shorten telomeres by oxidative damage [31, 75]. Hence, dysfunctional telomeres and mitochondria are the main actors of a vicious circle reducing cell fitness and promoting cellular aging [58]. Here we reveal UBQLN1 as an important physiological regulator linking mitochondria and telomere communication. Based on our results, either NAC or 5% O_2_ cultures reduces ROS, recovers mitochondria functions, and attenuates telomere shortening induced by *UBQLN1* deficiency. Yet, we recognize that telomere length was not recovered to that of WT cells, suggesting that additional role of *UBQLN1* in regulating telomere maintenance other than the damage produced by mitochondria oxidative stress.

We show that UBQLN1 plays important role in effective clearance of misfolded protein species and maintains proteostasis balance. UBQLN1 deficiency results in defective clearance and thus proteome imbalance, which in turn, causes defective mitochondria function and telomere damage and attrition. Our results are consistent with previous studies demonstrating that UBQLN1 binds to a variety of mitochondrial transmembrane proteins and is important for eliminating mislocalized mitochondrial proteins by proteasomal degradation, which is essential for the maintenance of mitochondria function [9, 10]. However, in these systems, loss of *UBQLN1* leads to cytosolic accumulation of mitochondrial proteins, which seems to be contradictory to our MASS result. Firstly, they analyzed cytosolic protein distinguished from mitochondria organelle but we used the total proteins for analysis (containing proteins locate in mitochondria) after *UBQLN1* knockout. Secondly, they used transient *UBQLN1* deletion cell line after 48 h of culture with doxycycline. Nevertheless, we obtained stable *UBQLN1* deficient cell line. We postulated that at the first step, *UBQLN1* deficiency leads to accumulation of mitochondrial protein (just as other proteins) in cytoplasm and subsequent mitochondria dysfunction. Disrupted mitochondria homeostasis leads to increased mitophagy and finally causes the reduced mitochondria components. Reasonable explanation for decrease of mitochondria components in *UBQLN1*^−/−^ cells in our experimental conditions is the increase of mitophagy, as supported by elevated mitophagy marker, LC3-II as well as reduced P62 (Fig. 4I).

The ubiquitin proteasome system is involved in the pathogenesis of various types of cancers and neurodegenerative diseases. Mutations in *UBQLN1* are associated with motor neuron diseases such as Alzheimer’s disease [12]. These mutations are thought to be pathogenic in part due to the accumulation of ubiquitinated protein aggregates and defects in proteasomal degradation [76]. In mice, Ubquilin-1 protects them from oxidative stress and ischemic stroke-caused neuronal injury through facilitating removal of damaged proteins [77, 78]. Impaired mitochondrial biogenesis also contributes to mitochondrial dysfunction in Alzheimer’s Disease [79]. We found that deficiency of *UBQLN1* in hESCs leads to impaired ubiquitin-proteasome system mediated protein degradation and dysfunctional mitochondria as well as elevated ROS level. We show that NAC boosts mitochondria biogenesis and attenuates telomere shortening in *UBQLN1*-deficient cells. NAC can inhibit mTOR signaling pathway, involved in hyperactive metabolisms [80]. Notably, NAC has been ascribed as a potential therapeutic intervention to ameliorate Alzheimer’s disease [81–83].

Mitochondria dysfunction in *UBQLN1*-deficient cells leads to an increase in mitophagy, which eliminates defective mitochondria via lysosomes [84], so the lysosomal activity increases at this time; On the other hand, intracellular protein accumulation itself can activate the lysosomal pathway, at least in our proteomics results, the autophagy pathway rises, but this stressful activation of the lysosomal pathway cannot remove all accumulated proteins. Likewise, MG132 treated cells also exhibit an increase in autophagy, but the protein accumulation is still present [85], suggesting that protein-level autophagy activation cannot clear all the proteins accumulated due to damaged ubiquitin-proteasome pathway. In consistency, lysosomal enzymes (increasing lysosomal acidity and thus improving enzyme activity) activated through ML-SA1 promote clearing part of the accumulated protein, alleviating the mitochondrial dysfunction caused by protein aggregates, and thus reducing telomere damage.

In addition to the already known roles of UBQLN1 in neurodegenerative disorder, our results probably predict the potential roles of UBQLN1 in neurogenesis. The exact underlying mechanism requires further exploration.

## Conclusions

In conclusion, UBQLN1 functions to scavenge ubiquitinated proteins, preventing their overloading mitochondria and elevated mitophagy as well as excessive ROS. UBQLN1 maintains mitochondria and telomeres by regulating proteostasis and plays critical role in neuro-ectoderm differentiation.

### Electronic supplementary material

Below is the link to the electronic supplementary material.

Supplementary Material 1

## Data Availability

The accession number for all the RNA sequencing data reported in this paper is NCBI GEO: GSE213094 (token: wlgluekaxvszxkn). The raw data of LC-MASS have been submitted to PRIDE under accession number PXD036634; The raw data of quantitative proteomic MASS have been submitted to PRIDE under accession number PXD036642.

## Resource availability

Further information and requests for resources and reagents should be directed to and will be fulfilled by the corresponding author, Lin Liu (liulin@nankai.edu.cn) and Baofa Sun (sunbf@nankai.edu.cn).
