# Supplementary figures and images for: UBQLN1 links proteostasis and mitochondria function to telomere maintenance in human embryonic stem cells
Source: Stem Cell Res Ther. 2024 Jun 21;15:180. doi: 10.1186/s13287-024-03789-y (PMC11191273; doi:10.1186/s13287-024-03789-y)

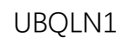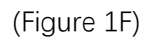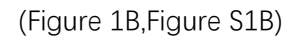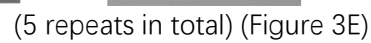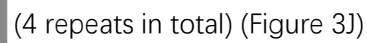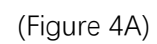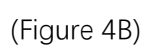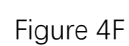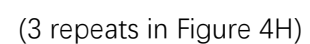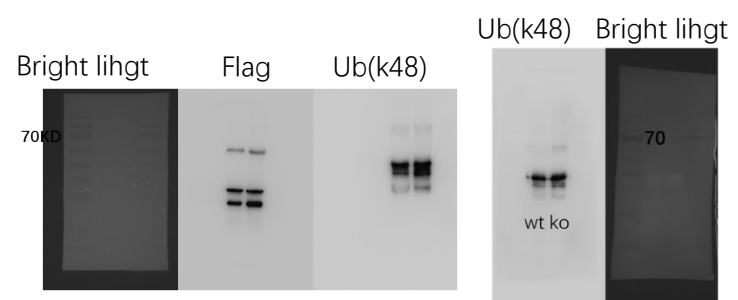

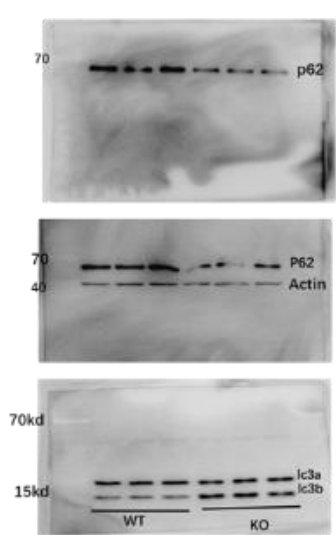

(Figure 4J)

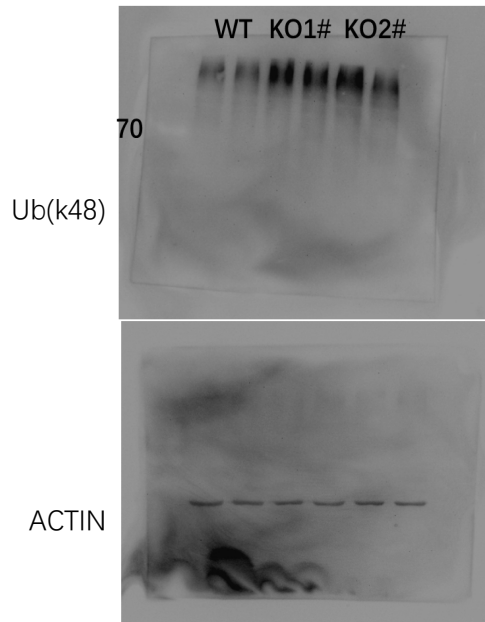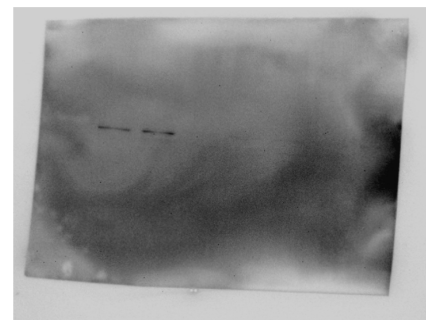

UBQLN1

(Figure 5A)

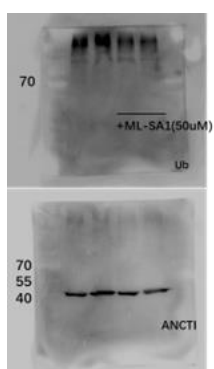

(Figure 5E)

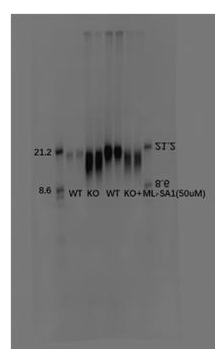

(Figure 5G)

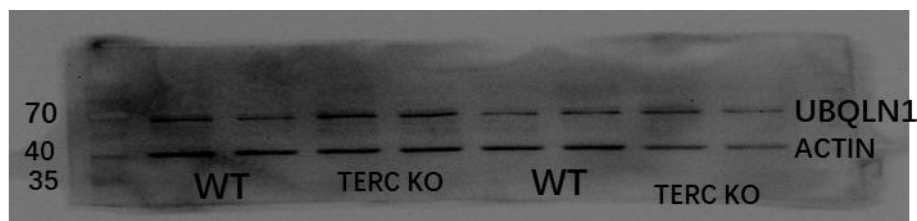

(Figure S1B))

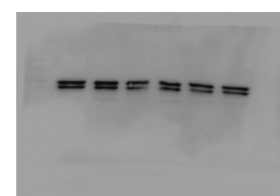

OCT4

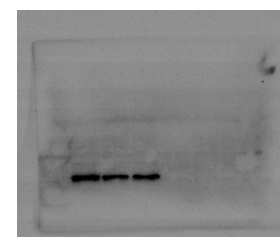

UBQLN1

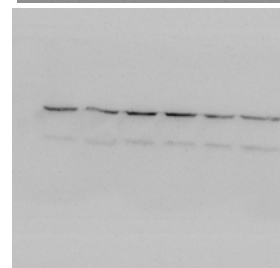

ACTIN

Figure S1E

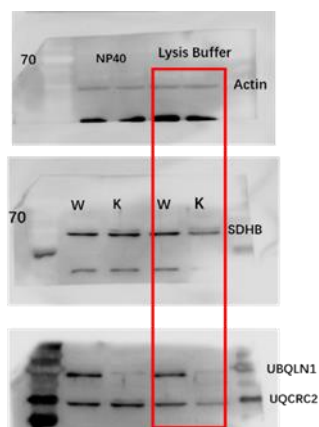

(Figure S3B)

Supplement: Supplementary file 1 — Supplementary Material 1 [file 13287_2024_3789_MOESM1_ESM.pdf]
